# Ultrasonic Modulation of the Technological and Functional Properties of Yeast Strains

**DOI:** 10.3390/microorganisms8091399

**Published:** 2020-09-11

**Authors:** Barbara Speranza, Daniela Campaniello, Clelia Altieri, Milena Sinigaglia, Antonio Bevilacqua, Maria Rosaria Corbo

**Affiliations:** Department of the Science of Agriculture, Food and Environment, University of Foggia, 71122 Foggia, Italy; barbara.speranza@unifg.it (B.S.); daniela.campaniello@unifg.it (D.C.); clelia.altieri@unifg.it (C.A.); milena.sinigaglia@unifg.it (M.S.)

**Keywords:** ultrasound, starter, yeasts, growth enhancement, gastrointestinal transit

## Abstract

This research was aimed at studying the effects of low intensity ultrasound (US) on some technological and functional properties of eight strains of *Saccharomyces cerevisiae*; namely, growth patterns (growth at 2–5% of NaCl or at 37 °C), autoaggregation and tolerance to simulated gastrointestinal conditions were evaluated. A US treatment was applied at 20% of net power (130 W) by a modulating duration (2–10 min) and pulses (2–10 s). The viable count (4.81–6.33 log CFU/mL) was not affected by US, while in terms of technological traits the effect was strain specific; in particular, for some strains a positive effect of US was found with a significant growth enhancement (growth index > 120%). The treatment was also able to increase the autoaggregation of some strains, thus suggesting that US could represent a promising way to treat and select nonconventional functional yeasts for food applications.

## 1. Introduction

Ultrasound (US) is a “green” technology which uses sonic waves (from 20 kHz to 10 MHz) widely applied in food processing, sonochemistry and medical diagnosis [1,2,3]. In general, high intensity and low frequency USs (intensity from 10 to 1000 W/cm^2^ and frequency from 20 to 100 kHz) are used in food processing as antimicrobial treatments, whereas low intensity and high frequency USs (intensity < 1 W/cm^2^; frequency > 100 kHz) are used for nondestructive applications, such as changing the physical and chemical structure of matrices (defoaming, deaeration, filtration, pickling, drying, defrosting, fractionation, accelerated wine aging, and extrusion) [3].

Regarding food applications, most studies performed on US have been initially addressed on the effects on cell destruction—high intensity USs (10–1000 W/cm^2^) have, in fact, a great effectiveness in terms of disrupting microbial cells, due to the power levels associated, which are high enough to generate cavitation and exert an antimicrobial effect [4].

However, in recent years, low intensity USs (less than 10 W/cm^2^) have been proposed as an innovative treatment to increase the yield of some biotechnological processes without affecting cell viability [1,5,6], since several studies have demonstrated that this form of US application produces a steady cavitation and provides repairable damages to cells, changing their living state and, consequently, accelerating their proliferation and metabolism [7,8]. For example, in food fermentation, the application of low intensity USs was found to improve the performances of microorganisms and the quality of the final products [9,10,11]. The literature reports some positive effects, such as: (a) the enhancement of the activity of different enzymes [7,12]; (b) a shortening of the fermentation time with a high reproducibility and accuracy of the process [13,14]; (c) the improvement of some technological properties of the end products [15]. Regarding bacteria, some positive effects include an enhanced biofilm formation for an increased transport of oxygen and nutrients to deeper layers of the microbial association [16,17], a lower acidification and postacidification of probiotics inoculated in functional beverages [17,18,19] and an increase in fermentation efficiency [20]. An improved growth of probiotics (e.g., *Bifidobacterium* sp.), associated to an increase in the lactose hydrolysis and transgalactosylation in milk, was also observed [21,22].

Most data are on US-treated bacteria, but promising positive applications on yeasts are also described in the literature, such as the growth enhancement of *Saccharomyces cerevisiae* [23], a better kinetics of fermentation both using pentose and hexose [24], a reduction in wine and beer fermentation time by 50%, as well as an increased yield of ethanol production and a reduction in dissolved CO_2_ [25].

However, these studies highlight a high variability and that the impact of US on yeasts’ performances depends on the type of microorganism, the growth phase (e.g., adaptation, exponential, etc.), the properties of the medium and the parameters of the treatment (intermittent or continuous application of US), thus it is difficult to find a general model.

This research was aimed at studying the effects of low intensity US on some technological and functional properties of eight strains of *Saccharomyces cerevisiae*; namely, growth patterns (growth at 2–5% of NaCl or at 37 °C), autoaggregation and tolerance to simulated gastrointestinal conditions were evaluated. The US treatment was applied at 20% of net power (130 W) by modulating the duration (2–10 min) and pulses (2–10 s).

## 2. Materials and Methods

### 2.1. Strains

Eight strains of *S. cerevisiae*, labelled with a numerical code (2, 4, 17, 41, 4y, WB, W21, WL43) and isolated from sourdough [26] or from Uva di Troia grapes [27], were used in this research. All strains belong to the Culture Collection of the Laboratory of Predictive Microbiology, University of Foggia; they were maintained at 4 °C on yeast–peptone–glucose-agar slants (YPG agar) (10 g/L yeast extract; 20 g/L bacteriological peptone; 20 g/L glucose; 15 g/L agar) and grown in YPG broth (25 °C for 48 h) before each assay. All media and ingredients were purchased from Oxoid (Basingstoke, UK).

### 2.2. Ultrasound Treatment (US)

Aliquots of 20 mL saline solution (9% NaCl w/V) were individually inoculated to ca. 5 log CFU/mL with each tested strain and treated by a VC 130 Vibra Cell Ultrasound device (Sonics and Materials Inc., Newtown, CT, USA: net power, 130 W). The combinations used for US processing are in Table 1; in particular, the treatment was applied at 20% of net power by modulating the duration (2–10 min) and the pulses (2–10 s). Before each treatment, the ultrasonic probe (5 × 60 mm; diameter × the active component of horn) was cleaned with ethanol and washed with sterile distilled water. After the treatment, the sample was immediately cooled in ice to avoid a potential thermal effect.

The viable count was measured before and after each treatment on YPG agar (25 °C for 48 h). A sterile saline solution inoculated with each strain but untreated through US was used as the control (A1).

### 2.3. Growth Assays

US-treated yeasts were inoculated in YPG broth to 3 log CFU/mL; the medium was supplemented with NaCl (2–5%-stress conditions for the strains used in this research) and incubated at 25 °C (yeast optimal temperature) or 37 °C (temperature of human body; probiotic properties for yeasts). Growth was evaluated after 24 and 48 h at an absorbance of 600 nm using a spectrophotometer UV–Vis DU 640 Beckman (Fullerton, CA, USA).

The results were modelled as the growth index [28] modified by Racioppo et al. [19]:

GI = Abss/Absc × 100

where Abs_s_ is the absorbance of US-treated strain and Abs_c_ is the absorbance of the control (untreated microorganism, combination A1).

GI was analyzed as suggested by Bevilacqua et al. [28] and modified as follows:

GI < 25%: complete inhibition;
25% < GI < 75%: partial inhibition;
GI > 75%: no inhibition.

A new class was added to point out a positive effect on yeast growth:

GI > 120%: growth enhancement.

### 2.4. Autoaggregation Assays

This assay was performed for all strains using a modified method reported by Bautista-Gallego et al. [29]. After growth at 25 °C for 48 h, 20 mL aliquots of yeast cultures were harvested by centrifugation and washed twice with PBS (phosphate saline buffer, 9 g/L NaCl and 0.30 g/L Na_2_HPO_4_·2H_2_O, Sigma-Aldrich, St. Louis, MO, USA). Afterwards, 2 mL of suspension was placed in sterile tubes containing 18 mL of PBS, and each tube was treated by US, according to Table 1. After the treatment, the absorbance at 600 nm of the upper suspension was monitored after 0 (*A*_0_) and 2 h at 25 °C (room temperature) (*A_t_*). The experiments were performed at least in duplicate. The autoaggregation percentage was calculated with the following formula [29]:

$$A\%=1-\left(\frac{A_{t}}{A_{0}}\right)\times 100$$

where *A_t_* and *A*_0_ are the absorbance at the time *t* (2 h) and the initial value, respectively.

### 2.5. Simulated Gastrointestinal Conditions

Tolerance to simulated gastrointestinal conditions was evaluated using the method reported by Petruzzi et al. [30]; the assay was performed only on *S. cerevisiae* 2 and 17, untreated or treated at 20% for 10 min and pulses at 10 s (combination D3). Three different solutions (SS, SGJ and SIF) were freshly prepared as follows:

1. SS, Salivary conditions, i.e., a sterile electrolyte solution (pH 6.5) containing 0.22 g/L CaCl_2_ (C. Erba, Milan, Italy), 6.5 g/L NaCl (C. Erba), 2.2 g/L KCl (J.T. Baker, Milan, Italy), 1.2 g/L NaHCO_3_ (Sigma-Aldrich), and 100 mg/L lysozyme (Sigma-Aldrich) [31].
2. SGJ, Simulated Gastric Juice, i.e., a saline solution (0.9% NaCl) containing 3 g/L pepsin (porcine gastric mucosal, Sigma-Aldrich) and buffered to pH 2 [32].
3. SIF, Simulated Intestinal Fluid, i.e., a fluid prepared by mixing 1 g/L of pancreatin (porcine pancreas, Sigma-Aldrich) and 3 g/L of bile extract (bile extract porcine, Sigma-Aldrich) in a solution (pH 8) containing 6.5 g/L NaCl, 0.835 g/L KCl, 0.22 g/L CaCl_2_, 1386 g/L NaHCO_3_) [32].

Prior to use, each solution was sterilized by filtering through membranes (0.20 μm pore size; Minisart, Sartorius, Goettingen, Germany). Yeasts were suspended (about 6.5–7 log CFU/mL) into SS, SGJ and SIF and incubated at 37 °C for 5, 120 and 240 min, respectively, on an orbital shaker (200 rpm). The viable count was determined before and after each phase on YPG agar (25 °C for 48 h).

A further assay was also performed through a sequential protocol (SS → SGJ → SIF); after each step, yeast cells were recovered by centrifugation at 13,500× *g*, 15 min at 25 °C to completely remove the broth and used to inoculate the subsequent solution.

### 2.6. Statistical Analysis

The experiments were performed at least on two independent samples; for each sample, the analyses were conducted twice. Significant differences were pointed out through a *t*-test (paired comparison) or one-way ANOVA (Analysis of Variance) and Tukey’s test (multiple comparison). The *p*-level was set to 0.05. Statistical analysis was conducted through the software Statistica for Windows, v12.0 (Statsoft, Tulsa, OK, USA).

## 3. Results

Table 2 reports the viable count (log CFU/mL) of US-treated *S. cerevisiae* strains, compared to the control (A1, untreated yeast): US treatment did not reduce yeasts cell count, which comprised between 5 ± 0.30 log CFU/mL (strain 4) and 5.73 ± 0.31 log CFU/mL (strain WB) in the control, against values of 5.16 ± 0.29 log CFU/mL (strain 4) and 5.73 ± 0.28 log CFU/mL (strain 41) recovered after the most drastic treatment (D3, 20% of the net power, 10 min, pulses at 10 s). Moreover, no significant differences were found among US treatments (*p* > 0.05).

After a US application, even if a microorganism does not lose its viability, its growth could be compromised resulting in it being delayed, inhibited or even enhanced; thus, to evaluate how US-treated yeasts were able to grow under different conditions, their profiles were studied during growth at 25 and 37 °C and in presence of NaCl (2–5%). The approach of the Growth Index (GI) was used, as suggested by Bevilacqua et al. [28] and Racioppo et al. [19]. In general, a GI > 75% suggests that a treatment does not affect the growth kinetic, a GI < 25% or in the range 25–75% highlights a strong or a partial inhibition, respectively, and GI > 120% indicates a growth enhancement.

**Figure 1:**
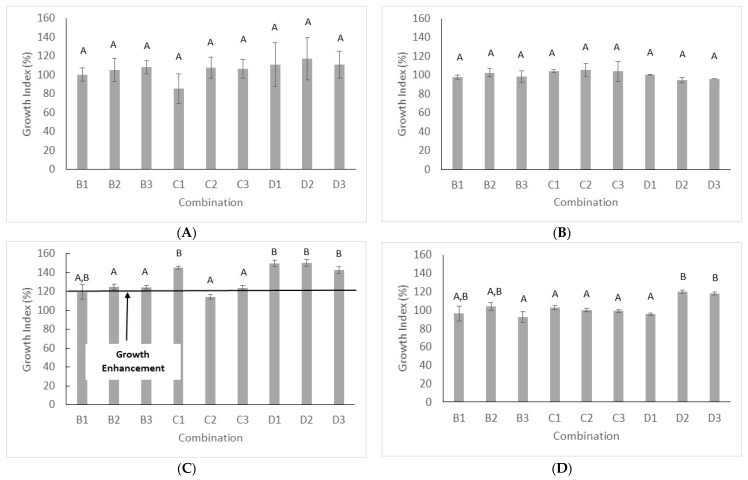
Growth Index (%) of US-treated *S. cerevisiae* 17 (**A**) and *S. cerevisiae* 41 (**B**) after 24 h in YPG incubated at 37 °C and *S. cerevisiae* 4y (**C**) and *S. cerevisiae* WB (**D**) after 48 h in YPG with 5% NaCl added incubated at 25 °C. The growth index was evaluated as the ratio absorbance of treated yeasts vs. absorbance of untreated yeasts (combination A1). Mean values ± standard deviation. Letters indicate significant differences (one-way ANOVA and Tukey’s test, *p* < 0.05).

Growth was evaluated either after 24 and 48 h; generally, yeast growth after 24 h was stunted, thus GI evaluation could lead to artefacts. However, some strains experienced a significant growth (OD at least 0.6–0.8), therefore, for these microorganisms, GI was evaluated (strains 17, 41, 4y and WB). Figure 1A,B show the GI of the strains 17 and 41; GI was in the range 100–120% (growth similar to the untreated sample, combination A1) and comparable results were found for the strains 4y and WB. However, for these last strains, the GI in presence of salt revealed a positive effect of US, with a GI > 120% (growth enhancement). This effect was found in the combination B1, D1, D2, and D3 (Figure 1C) for the strains 4y and D2, and D3 for the strain WB (Figure 1D).

Table 3 shows yeasts’ GI after 48 h; generally, US treatment did not exert a negative effect on yeast growth at 25, 37 °C or in presence of 2% salt, because GI was >75%. However, some strains experienced a GI > 120% in some combinations and for some assays, that is:

(a) Strain 17 in the combinations B2, B3 and C1 at 37 °C, with a GI ranging from 132.19 to 159.59%
(b) Strain 41 at 37 °C in the combinations C1 (GI, 233%) and C2 (GI, 185.71%).

Once the effects of US on technological properties was studied, a second step focusing on two main functional yeast characters, i.e., autoaggregation and survival during gastrointestinal transit, was performed.

**Figure 2:**
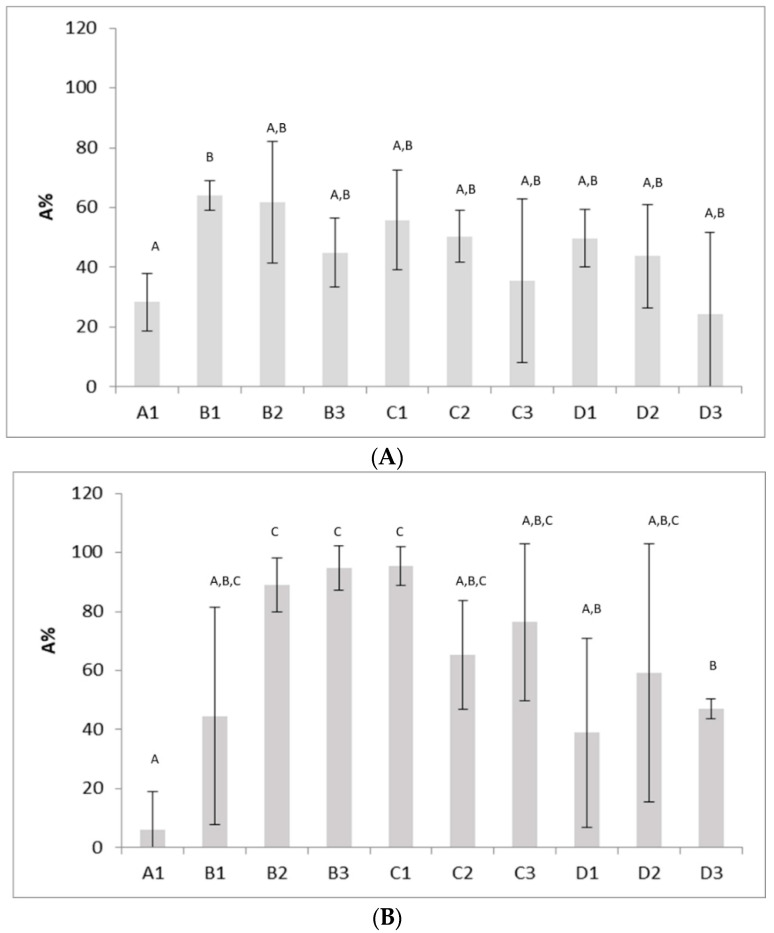
Autoaggregation percentage (A%) of *S. cerevisiae* 2 (**A**) and *S. cerevisiae* 17 (**B**), 2 h after the US treatment. Mean values ± standard deviation. A1, untreated yeast. The letters indicate significant differences (one-way ANOVA and Tukey’s test, *p* < 0.05).

As known, autoaggregation is an indirect tool to assess the ability of microorganisms to adhere to gut mucosa [33,34,35,36]. Figure 2 shows the effects of US on autoaggregation for *S. cerevisiae* 2 (A) and *S. cerevisiae* 17 (B) which were the yeasts showing the most interesting results. In general, US treatments did not affect this property for most yeasts, recovering autoaggregation values of 30–40%. However, for *S. cerevisiae* 2 and *S. cerevisiae* 17, the capacity to form aggregates was improved by US application—*S. cerevisiae* 2 experienced an increase in autoaggregation from 30 (combination A1) to 63% (combination B1), while for strain 17, autoaggregation values were 94.69 (±7.51%) and 95.32% (±6.62%), respectively, against an autoaggregation of 5.95% (±13%) measured for the untreated yeast (A1) (Figure 2B). The autoaggregation of other strains was not affected.

In the last step, the tolerance of US-treated yeast strains to simulated gastrointestinal conditions was evaluated by first testing each phase separately (salivary condition, gastric condition, intestinal condition), and then, in a sequential protocol; only the effect of the most drastic treatment (combination D3) was evaluated.

The test strain *S. cerevisiae* 17 was used because of the positive effect exerted by US on both growth patterns and autoaggregation, and so *S. cerevisiae* strain 2 was used as a control because in the past it was characterized as a potential probiotic strain able to survive the transit into the gut [26,37]. US did not affect viability both in the phases studied alone and in the sequential experiment (*p* > 0.05) and this result is of concern, because it suggests that a preliminary treatment with US does not exert a negative effect of yeast viability even if they experience a harsh environment such as the gastrointestinal tract (Table 4).

## 4. Discussion

To date, most studies have shown the possibility to use low intensity US for positive applications such as the inactivation/enhancement of some enzymes, extraction of bioactive compounds from different sources, modulation of microbial metabolism, enhancement of biofilm formation, and other useful food applications such as changing the physical and chemical structure of matrices [3,38,39]. Nevertheless, although the application of low intensity US is gaining more and more consensus in food processing, conflicting results were often recovered and finding a unifying behavior or conclusion about the effects of US on yeasts appears almost impossible. For example, when applied on *S. cerevisiae* cells, US was found to promote a growth acceleration by Lanchun and coworkers [23], but also a growth reduction. Some studies showed positive effects [40,41]; a 4-fold increase in ethanol productivity was found when US (35 kHz, 1.48 W/cm^2^) was applied on *S. cerevisiae* MTCC 170 [40], but negative effects on yeast performances have been recovered by Huezo et al. [42], using either direct US (at different intensities ranging from 23 to 32 W/L) or indirect US (1.4 W/L intensity). Besides the contradictory results, some aspects have been never explored, such as the ultrasonic effects on the technological and functional properties of potential functional yeasts; in fact, an open question remains on the possibility to use US as a promising way to improve yeast growth in suboptimal conditions (more salt or higher temperature) and to increase their functional properties such as autoaggregation and tolerance to simulated gastrointestinal conditions. In this research, a US treatment was applied at 20% of net power (130 W) by a modulating duration (2–10 min) and pulses (2–10 s) on eight strains of *S. cerevisiae,* isolated from sourdough [26] and from Uva di Troia grapes [27] for their functional traits. In particular, four *S. cerevisiae* strains (2, 4, 17 and 41) were selected as they showed an advantageous survival during transit into the gut, had high resistance to pH and salt, as well as a good ability to grow in a wide range of temperatures; moreover, they showed very interesting probiotic traits in terms of biofilm formation, hydrophobicity and survival at pH 2.5 and with bile salts added [26], while *S. cerevisiae* 4y, WB, W21, WL43 were selected as promising functional strains due to their ability to remove ochratoxin A (OTA) in the gut (unpublished results).

When a US treatment is applied, especially on potentially probiotic strains, the first aspect to be evaluated is the effect on viability. The results obtained showed that the treatments tested in this research never reduced yeast viability, even after the most drastic treatment.

However, after a US treatment, a microorganism, even if viable, could be damaged and thus present a delay or an inhibition in its growth. To evaluate how US-treated yeasts grow under nonoptimal conditions, the Growth Index (GI) approach [19,28] was used: both at 37 °C and in presence of 2% salt, US treatment did not affect growth. On the other hand, for four strains (*S. cerevisiae* 4, 17, 41 and WB) a significant growth enhancement was found, due probably to a potential improved membrane permeability. In fact, numerous evidence on bacteria indicate that low intensity US can stimulate growth and metabolic changes for a facilitated transportation of molecules across the cell membrane and an accelerated exchange of substances [43,44,45,46]. Wu et al. [47] demonstrated that an ultrasonic application on yeasts first caused the breakdown of the cell wall, then the cell membrane was weakened by thinning, causing the release of polysaccharides from the cell wall and intracellular proteins from the membrane. These effects are due to cavitation and to the formation of micromechanical shock waves which, associated with changes in temperature (up to 5 °C) and pressure (1 atm), are able to alter the membrane structure and its functional components [48,49,50]. In 2017, the mechanism of sonoporation (i.e., the formation of transient cavities or pores on cell membranes from cavitation) has been suggested by Ojha and coworkers [11] who explained the effect of US on cells as the result of at least six different mechanisms (cavitation, push, pull, jetting, streaming and translation) which can act alone or together [3].

Growth enhancement of US on yeasts was found in the past by several authors for other purposes. Dai et al. [8] found that a low intensity treatment could improve biomass production by *S. cerevisiae* (yield increased by 127%) and ethanol production. They attributed these effects to at least two phenomena: the decrease in CO_2_ content in the liquid media with a positive effect on yeast metabolism and the increase in cell permeability, as evidenced by an increased ratio of proteins and polysaccharides in the broth. However, some factors, such as yeast size, floccules, surface charges and to some extent composition, could change as a function of species and strains and this could be related to the different effect of US on the strains used in this research, although they belonged to the same species.

The second step of this study was on the effects of US on two main functional yeast characters, i.e., autoaggregation and survival to gastrointestinal transit. In general, US treatments did not affect autoaggregation for most yeasts, but for *S. cerevisiae* 2 and *S. cerevisiae* 17, this capacity was increased by a US application.

The increase in autoaggregation as a consequence of US is a desirable property, because aggregates can increase the microbial adherence to the intestine, thus providing advantages in the colonization of the GI tract [51], as well as the ability to survive in harsh environments [52]. This result was already observed by Bevilacqua et al. [17] and Racioppo et al. [19] for the application of US on bacteria and it is a very promising effect, since a higher autoaggregation is related to an increased adhesion in the gut (adhesion to mucus) [34], although this effect should be confirmed by other assays, such as biofilm formation and adhesion to intestinal model cell lines.

The positive effect of US on autoaggregation could be the result of a clumping effect reported in the past at least on bacteria. Tabatabaie and Mortazavi [53] studied the effect of sonic waves on *Lactobacillus acidophilus*, *Lacticaseibacillus casei* and *Lactococcus lactis* subsp. *cremoris*, and found that US induced an increase in the ability to autoaggregate in lactobacilli, and form “streptobacillus”-like clumps with an enhanced adhesion.

Autoaggregation, or flocculation, depends on several factors, such as cell surface properties, the presence of Ca^2+^ or mannose in the medium [54], culture replicative age [55]. It is mediated by cell-surface molecules; thus, it is strongly affected by the different cell wall composition of each strain and the presence of appendages protruding from the wall [54]. This may explain the differences found on the effect of US of autoaggregation of different strains.

Finally, US did not affect yeasts’ tolerance to simulated gastrointestinal conditions; this is another crucial aspect for probiotic yeasts, since a probiotic strain should remain viable after facing the digestive tract conditions and reach the colon where it should proliferate, exerting its beneficial probiotic effects [56,57,58].

In conclusion, US could represent a promising way to improve some technological and functional properties of *S. cerevisiae* strains. In fact, a low intensity US treatment was able to enhance the growth of *S. cerevisiae* at 37 °C (strains 4y and WB) and in the presence of 2% salt (strains 17 and 41). The treatment could modulate the metabolism of yeasts: in fact, an increase in the autoaggregation capacity was recovered for *S. cerevisiae* 2 and 17. However, since a strain-dependence was found, further investigations are required to understand the mechanisms behind the recovered ultrasonic modulation of yeast properties.

## Figures and Tables

**Table 1:** Combinations of power, duration of the treatment and pulse.

	Power (%)	Time (min)	Pulse (s)
A1	0	0	0
B1	20	2	2
B2	20	2	6
B3	20	2	10
C1	20	6	2
C2	20	6	6
C3	20	6	10
D1	20	10	2
D2	20	10	6
D3	20	10	10

**Table 2:** Viable count (log CFU/mL) of US-treated *Saccharomyces cerevisiae* strains, compared to the control (untreated microorganism, A1). Mean values ± standard deviation. ns, for each strain the differences were not significant (one-way ANOVA and Tukey’s test, *p* > 0.05).

Strains	Combinations										
	A1	B1	B2	B3	C1	C2	C3	D1	D2	D3	
2	5.55 ± 0.05	5.64 ± 0.02	5.67 ± 0.08	5.70 ± 0	5.68 ± 0	5.77 ± 0.10	5.76 ± 0.06	5.69 ± 0.17	5.65 ± 0.11	5.71 ± 0.01	ns
4	5 ± 0.30	4.98 ± 0.32	5 ± 0.35	4.98 ± 0.31	4.81 ± 0.30	4.95 ± 0.30	5.22 ± 0.34	5.19 ± 0.30	5 ± 0.32	5.16 ± 0.29	ns
17	5.68 ± 0.26	5.90 ± 0.20	5.79 ± 0	5.78 ± 0.06	5.82 ± 0.10	5.89 ± 0.27	5.76 ± 0.21	5.83 ± 0.07	5.78 ± 0.25	6.07 ± 0.36	ns
41	5.64 ± 0.15	6.02 ± 0.12	5.72 ± 0.38	5.48 ± 0.33	5.66 ± 0.30	5.55 ± 0.33	5.57 ± 0.35	5.63 ± 0.33	5.65 ± 0.34	5.73 ± 0.28	ns
4y	5.54 ± 0.14	5.51 ± 0.30	5.42 ± 0.35	5.52 ± 0.32	5.49 ± 0.32	5.48 ± 0.31	5.39 ± 0.34	5.40 ± 0.31	5.44 ± 0.30	5.49 ± 0.29	ns
WB	5.73 ± 0.31	5.67 ± 0.33	5.68 ± 0.35	4.81 ± 0.33	4.81 ± 0.33	5.52 ± 0.30	5.53 ± 0.34	5.41 ± 0.35	5.43 ± 0.33	5.66 ± 0.35	ns
W21	5.49 ± 0.21	5.49 ± 0.21	5.53 ± 0.28	5.28 ± 0.30	5.48 ± 0.29	5.64 ± 0.30	6.19 ± 0.31	5.58 ± 0.34	5.46 ± 0.35	5.56 ± 0.27	ns
WL43	5.70 ± 0.21	5.48 ± 0.20	5.81 ± 0.25	5.59 ± 0.23	5.68 ± 0.27	5.50 ± 0.22	5.18 ± 0.33	5.58 ± 0.27	5.45 ± 0.35	5.56 ± 0.20	ns

**Table 3:** Growth Index (%) of US-treated strains (*S. cerevisiae* 2, 4, 17, 41, 4y, WB, W21 and WL43) in YPG broth incubated at 25 and 37 °C or in presence of 2% NaCl after 48 h. The growth index was evaluated as the ratio absorbance of treated yeasts vs. absorbance of untreated yeasts (combination A1). Mean values ± standard deviation. The letters indicate significant differences in a line (one-way ANOVA and Tukey’s test, *p* < 0.05).

Combinations	B1	B2	B3	C1	C2	C3	D1	D2	D3
	**Strain 2**								
25 °C	70.85 ± 13.82A	65.26 ± 7.85A	67.66 ± 26.79A	64.21 ± 17.45A	72.16 ± 23.98A	84.91 ± 11.19A	66.73 ± 34.87A	69.31 ± 16.34A	82.11 ± 30.71A
37 °C	87.30 ± 10.41A	98.98 ± 1.27A	101.31 ± 11.13A	104.80 ± 10.06A	94.18 ± 14.72A	115.85 ± 7.59A	108.61 ± 18.88A	129.33 ± 13.97A,B	90.30 ± 2.67A
2% NaCl	129.17 ± 0C	129.76 ± 0C	84.52 ± 0A	106.55 ± 0B	106.55 ± 0B	80.65 ± 9.68A	93.75 ± 8.84A,B	109.82 ± 14.73A,B,C	85.42 ± 2.10A
	**Strain 4**								
25 °C	89.64 ± 3.50A	98.54 ± 8.50A	94.31 ± 9.42A	105.73 ± 2.01A	101.95 ± 0.34A	105.48 ± 5.92A	103.66 ± 6.43A	106.86 ± 1.44A	108.16 ± 1.09A
37 °C	100.93 ± 7.45A	104.65 ± 3.16A	103.48 ± 1.50A	97.20 ± 1.81A	100.57 ± 10.89A	99.34 ± 7.36A	101.51 ± 0.57A	97.28 ± 2.10A	98.61 ± 2.36A
2% NaCl	106.29 ± 11.31A	117.64 ± 12.51A	124.72 ± 13.27A	108.52 ± 11.54A	120.06 ± 12.77A	121.13 ± 12.88A	117.54 ± 12.50A	132.38 ± 14.08A	131.70 ± 14.01A
	**Strain 17**								
25 °C	117.95 ± 24.52A	113.54 ± 33.88A	131.62 ± 20.05A	123.28 ± 17.44A	130.19 ± 14.21A	119.73 ± 31.37A	110.59 ± 44.45A	104.93 ± 25.55A	104.30 ± 13.38A
37 °C	103.81 ± 20.15 A,B	159.59 ± 20.49B	132.19 ± 9.47B	133.44 ± 1.75B	104.96 ± 9.63 A	97.85 ± 11.63 A	98.71 ± 11.71 A	97.90 ± 16.48 A	90.03 ± 11.61 A
2% NaCl	111.46 ± 16.20A	137.59 ± 21.00A	125.93 ± 28.08A	136.73 ± 25.65A	134.27 ± 16.30A	113.90 ± 21.86A	133.17 ± 3.46A	106.61 ± 24.29A	146.80 ± 12.41A
	**Strain 41**								
25 °C	91.48 ± 45.95A	140.80 ± 21.78A	138.89 ± 26.72A	132.81 ± 30.71A	110.69 ± 15.75A	88.45 ± 201.64A	111.94 ± 87.35A	87.82 ± 0.48A	149.82 ± 48.96A
37 °C	133.68 ± 12.76A	127.11 ± 8.15A	132.85 ± 1.97A	233.01 ± 11.63C	185.71 ± 25.08B	112.99 ± 35.86A	100.64 ± 33.34A	137.91 ± 5.18A	96.63 ± 35.94A
2% NaCl	92.04 ± 3.12C	73.48 ± 9.64A,B	72.37 ± 13.43A,B	83.67 ± 12.23A,B	67.58 ± 2.99A	75.51 ± 3.93B	90.73 ± 2.55C	87.55 ± 13.31A,B	102.76 ± 0.24D
	**Strain 4y**								
25 °C	111.42 ± 31.77A	116.27 ± 57.24A	149.59 ± 17.92A	108.11 ± 33.34A	93.04 ± 38.29A	91.97 ± 37.85A	79.81 ± 32.84A	142.75 ± 58.75A	171.35 ± 70.52A
37 °C	94.15 ± 26.00A	102.40 ± 7.63A	94.84 ± 10.43A	98.99 ± 3.55A	96.69 ± 3.47A	95.37 ± 3.42A	105.73 ± 3.79A	100.17 ± 3.59A	97.11 ± 3.48A
2% NaCl	114.68 ± 18.47A	110.90 ± 23.38A	101.60 ± 4.89A	118.40 ± 17.69A	120.78 ± 18.05A	113.91 ± 17.02A	114.29 ± 17.08A	117.61 ± 17.51A	138.62 ± 20.71A
	**Strain WB**								
25 °C	144.23 ± 66.79A	134.73 ± 60.40A	101.09 ± 28.64A	155.38 ± 45.86A	167.74 ± 49.50A	123.34 ± 36.40A	201.03 ± 59.33A	215.41 ± 63.57A	275.44 ± 81.29A
37 °C	90.46 ± 3.88A	99.75 ± 2.72A	98.31 ± 5.18A	100.31 ± 3.59A	96.57 ± 3.46A	90.35 ± 3.23A	90.90 ± 3.25A	92.89 ± 3.33A	89.99 ± 3.22A
2% NaCl	112.17 ± 1.45A	105.95 ± 18.73A	112.64 ± 11.17A	121.05 ± 13.48A	87.42 ± 9.73A	122.17 ± 13.60A	115.49 ± 12.86A	128.00 ± 14.25A	120.12 ± 14.38A
	**Strain W21**								
25 °C	81.72 ± 13.80A	102.02 ± 32.70A	59.60 ± 51.48A	109.05 ± 33.59A	93.83 ± 33.46A	105.93 ± 23.20A	106.78 ± 33.29A	93.70 ± 33.33A	102.57 ± 23.28A
37 °C	91.80 ± 13.68A	82.02 ± 22.62A	93.54 ± 25.11A	60.34 ± 43.19A	89.78 ± 23.36A	86.23 ± 33.23A	89.38 ± 43.20A	86.13 ± 23.30A	83.48 ± 43.12A
2% NaCl	97.30 ± 28.70A	97.82 ± 25.20A	101.58 ± 19.90A	102.04 ± 23.14A	98.95 ± 18.22A	97.56 ± 35.00A	95.45 ± 31.83A	100 ± 38.70A	98.95 ± 40.13A
	**Strain WL43**								
25 °C	101.38 ± 23.50A	87.26 ± 12.90A	86.30 ± 41.88A	88.46 ± 30.50A	95.07 ± 30.40A	84.44 ± 33.25A	92.25 ± 38.89A	93.15 ± 33.35A	92.97 ± 37.28A
37 °C	113.28 ± 18.60A	93.33 ± 27.42A	83.59 ± 35.19A	86.30 ± 40.89A	71.61 ± 28.33A	86.25 ± 31.20A	83.18 ± 23.20A	75.74 ± 13.90A	83.85 ± 40.10A
2% NaCl	106.75 ± 25.50A	110.97 ± 28.80A	102.27 ± 29.77A	107.72 ± 33.15A	108.96 ± 18.20A	113.37 ± 30A	99.68 ± 30.87A	105.52 ± 28.50A	104.35 ± 44.33A

**Table 4:** Viable count of US-treated *S. cerevisiae* 2 and *S. cerevisiae* 17 after simulated gastrointestinal transit. Mean values ± standard deviation. A1, untreated microorganism; D3, 20% of the net power, 10 min, pulses every 10 s (the more drastic treatment). ns, for each strain and step the differences between A1 (control) and US-treated combination (D3) were not significant (*t*-student’s test, *p* > 0.05).

Yeast	Treatment	Inoculum	Salivary Conditions	Gastric Conditions	Intestinal Conditions	Sequential Transit
*S. cerevisiae* 2	A1	6.40 ± 0.30	6.54 ± 0.29	6.78 ± 0.10	6.78 ± 0.08	5.81 ± 0.50
	D3	6.78 ± 0.14	6.64 ± 0.30	6.34 ± 0.32	6.48 ± 0.33	6.32 ± 0.18
		ns	ns	ns	ns	ns
*S. cerevisiae* 17	A1	7.02 ± 0.35	7.16 ± 0.36	6.51 ± 0.33	6.50 ± 0.30	6.37 ± 0.25
	D3	6.54 ± 0.33	6.54 ± 0.35	6.45 ± 0.31	6.46 ± 0.36	6.23 ± 0.40
		ns	ns	ns	ns	ns
